# Leukocyte-Poor Platelet-Rich Plasma for the Management of Knee Osteoarthritis: A Retrospective Study With 12 Months of Follow-Up

**DOI:** 10.7759/cureus.69662

**Published:** 2024-09-18

**Authors:** Ashim Gupta, Arun Viswanath, G. Hari Kumar

**Affiliations:** 1 Regenerative Medicine, Future Biologics, Lawrenceville, USA; 2 Orthopedics, SP Fort Hospital, Thiruvananthapuram, IND

**Keywords:** autologous peripheral blood-derived orthobiologics, knee osteoarthritis, leukocyte-poor platelet rich plasma, lp prp, patient reported outcome measures, platelet-rich plasma, proms, prp, regenerative medicine

## Abstract

Introduction

The knee, the most frequently affected joint in osteoarthritis (OA), impacts the life quality of millions of individuals globally, resulting in a considerable healthcare burden. Conservative treatments are preferred, turning to surgical intervention when necessary. Nonetheless, these conventional modalities have drawbacks. Recently, the use of regenerative medicine therapies, including autologous peripheral blood-derived orthobiologics (APBOs), such as leukocyte-poor platelet-rich plasma (LP-PRP), has evolved and demonstrated the ability to manage knee OA. The primary objective of this investigation was to evaluate the efficacy of LP-PRP via widely used patient-reported outcome measures (PROMs) in grade I or II (on the Kellgren-Lawrence scale) knee OA patients. The secondary objective was to characterize the formulated LP-PRP and determine the efficiency of the leukodepletion filter used for leukocyte removal and platelet recovery.

Methods

This investigation was a retrospective analysis of data collected from patients treated at a single center over a period of 15 months. Data from 40 patients included in this study were intra-articularly injected with 3mL of formulated LP-PRP under ultrasound guidance. PROMs questionnaires, including Kujala and Western Ontario and McMaster Universities Osteoarthritis Index (WOMAC) scores, were used and responses were documented at baseline and up to 12 months follow-up. The characterization of the formulated LP-PRP and the efficiency of the leukodepletion filter in removing leukocytes and recovering platelets were assessed via complete blood count (CBC) analysis.

Results

The intra-articular administration of LP-PRP resulted in statistically significant improvements in Kujala and WOMAC scores in patients with Grade I or II OA of the knee at all follow-up time points (four to 12 months) compared to the respective baseline scores. The subgroup analysis showed significant improvements in Kujala and WOMAC scores in both male and female grade I or II knee OA patients with or without comorbidities, including diabetes and/or hypertension. The characterization of formulated PRP showed platelet concentration to be at least 6x compared to the baseline whole blood levels, the absolute platelet count to be at least 5 billion, and total leukocytes, lymphocytes, neutrophils, and RBCs were depleted by over 88%, 82%, 98%, and 98%, respectively. In addition, the utilization of the PuriBlood leukocyte reduction filter (Puriblood Medical Co. Ltd., Baoshan Township, Taiwan) led to the depletion of approximately 93% of leukocytes and the recovery of about 83% of platelets.

Conclusions

Administration of LP-PRP resulted in significant improvements in pain and function of patients suffering from grade I or II OA of the knee. In addition, the leukodepletion filter used to formulate LP-PRP, successfully resulted in the depletion of leukocytes while recovering the platelets. More sufficiently powered, multi-center, prospective, non-randomized, and randomized controlled trials with long-term follow-up are needed to further establish the effectiveness of this formulation in knee OA patients.

## Introduction

Osteoarthritis (OA) of the knee, a chronic inflammatory disorder, resulting in degeneration of the cartilage in the joint, is frequently accompanied by constant pain and stiffness, resulting in diminished mobility and quality of life (QoL) [1,2]. OA continues to be the most common progressive and degenerative joint illness, affecting nearly 7% of adults worldwide [3]. The increase in the incidence of OA is attributable to factors, such as aging and a rise in poor metabolic conditions, predominantly obesity [4]. Currently, knee OA is treated using non-pharmacological tactics such as weight management and physical therapy, nutraceuticals such as chondroitin, glucosamine, and undenatured type II collagen; pharmacological agents such as corticosteroids, opioids, viscosupplementation and non-steroidal anti-inflammatory drugs (NSAIDs); minimally invasive interventions such as genicular nerve radiofrequency ablation; and surgery, when the conventional therapies have been unresponsive or in advanced stages of OA [5-7]. However, these above-said treatment options have shortcomings, trying to provide symptomatic pain relief but not stopping the progression of OA and/or modifying the disease [5-7].

Recently, the use of regenerative medicine therapies, such as autologous peripheral blood-derived orthobiologics (APBOs), for managing knee OA has grown [7-11]. Platelet-rich plasma (PRP) is the most frequently utilized APBO, and several level I and II studies have shown its safety and effectiveness [12,13]. Notwithstanding the immense beneficial capability of PRP, quite a few issues remain. Particularly, various methods for the formulation of PRP exist, yielding differing compositions and characteristics [14]. In addition, inadequate information is available about the most apt PRP preparation for a specific indication [14]. Here, we concentrate on the leukocyte's presence, a highly debated aspect associated with the effectiveness of PRP and an essential discriminant in classifying PRP preparations. Several in vitro and pre-clinical studies have raised apprehensions related to the pro-inflammatory character of leukocytes found in the PRP preparations, attributed to their ability to activate the catabolic cascades, resulting in higher inflammatory cytokines expression, such as interleukin -1 (IL-1) and tumor necrosis factor-alpha (TNF-α) [14]. Moreover, subgroup analysis from recent level I studies has demonstrated superior efficacy of leukocyte poor (LP) PRP in terms of relieving pain (on Visual Analogue Scale score) and improving function (on International Knee Documentation Committee score) compared to the leukocyte rich (LR) PRP [12,15].

Traditionally, LP-PRP is formulated by using either differential centrifugation methods or the use of leukodepletion filters [16-18]. Herein, we intend to use a commercially available, patented (US11833289B2) leukodepletion filter (PuriBlood, Puriblood Medical Co. Ltd., Baoshan Township, Taiwan). The primary aim of this study was to assess the efficacy of LP-PRP via widely used patient-reported outcome measures (PROMs) in grade I or II (on the Kellgren-Lawrence scale) knee OA patients. The secondary objective was to characterize the formulated LP-PRP and determine the efficiency of the leukodepletion filter used for leukocyte removal and platelet recovery. We hypothesized that a leukodepletion filter can significantly remove leukocytes and recover platelets; intra-articular administration of formulated LP-PRP is efficacious in terms of improving pain and function in patients with grade I or II OA of the knee.

## Materials and methods

Study design

This investigation is a retrospective analysis of data collected from patients treated at a single center, SP Fort Hospital (Thiruvananthapuram, Kerala, India) from August 31, 2022, to October 13, 2023. This study is approved by the Institutional Ethics Committee, Sri Lakshmi Narayana Institute of Medical Sciences, Puducherry, India, with approval number IEC/C-01/2024, dated June 25, 2024. The participating patients provided informed consent for data to be gathered and evaluated for publication in a peer-reviewed journal, while no protected health information (PHI) was collected. Forty patients (both males and females) aged 20-75 years old diagnosed with grades I and II OA of the knee (on the Kellgren-Lawrence scale) were included. Patients <20 years old or >75 years old and diagnosed with grade III or IV knee OA were excluded. Outcome measures included Western Ontario and McMaster Universities Osteoarthritis Index (WOMAC) (0-96 scale, 0 represents the best health status and 96 the worst possible status) and Kujala Score (Anterior Knee Pain Scale, AKPS) (0-100 scale, higher score indicates better function), and were assessed at baseline and up to 12 months follow-up post-injection.

Preparation and characterization of leukocyte-poor platelet-rich plasma (LP-PRP)

**Figure 1:**
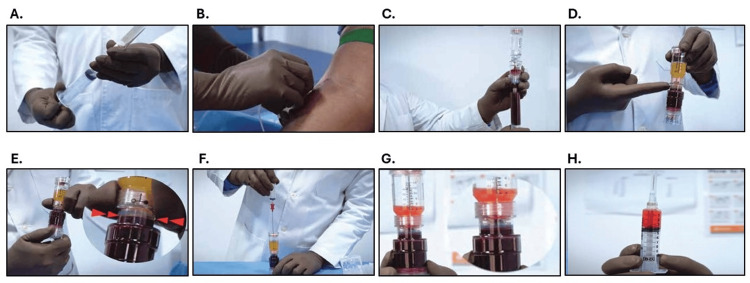
Steps involved in preparation of platelet-rich plasma (PRP) using TriCell TriPReP TUBEX®. (A) Fill syringe with Anticoagulant Citrate Dextrose Solution, Solution A, (B) blood withdrawal from antecubital vein, (C) transfer withdrawn blood in tube, (D) separation of whole blood into three layers (plasma, buffy coat, and red blood cells) after first spin, (E) separation of upper layers (plasma and buffy coat) and lower layer (red blood cells) by twisting the chamber clockwise, (F) withdrawal of supernatant platelet poor plasma after second spin, (G) suspension of platelets in residual plasma, and (H) resultant PRP.

Venipuncture was performed on all enrolled patients, i.e., 25 mL of venous blood (from an antecubital vein) was drawn in a syringe with 2 mL of anticoagulant (Anticoagulant Citrate Dextrose Solution, Solution A). The withdrawn blood was then transferred into a 25 mL sterile TriCell TriPReP TUBEX® (TriCell Biologics, Chennai, Tamil Nadu, India). The tubes were then centrifuged (Medico Plus centrifuge, REMI, Mumbai, Maharashtra, India) at 1288 g for 12 minutes to separate whole blood into three layers: plasma, buffy coat, and red blood cells (RBCs). Afterward, the upper layers in the tube (plasma and buffy coat) were separated from the lower layer (RBCs) by locking the middle part of the tube (by twisting the chamber clockwise). The tube was then centrifuged again at 1848 g for 5 minutes. Approximately 10 mL of the supernatant platelet-poor plasma (PPP) was aspirated and discarded, and the remaining plasma was shivered to suspend the settled platelets to obtain 3 mL PRP (Figure 1). This PRP was then collected from the tube via syringe and passed through a PuriBlood leukocyte reduction filter (Puriblood Medical Co. Ltd., Taiwan) to obtain LP-PRP.

For characterization, randomly selected samples of the patient’s whole blood, formulated PRP, and LP-PRP were sent for complete blood count (CBC) analysis to determine the number of platelets, white blood cells (WBCs)/total leukocytes, lymphocytes, neutrophils, and RBCs. In addition, multiple samples were tested to determine the effect of the PuriBlood leukocyte reduction filter (Puriblood Medical Co. Ltd., Taiwan) on the removal of WBCs and recovery of platelets.

Leukocyte-poor platelet-rich plasma (LP-PRP) injection

The patients are placed in a supine position with their knees bent at a 45° angle. The formulated LP-PRP was administered intra-articularly via infero-medial or infero-lateral method under ultrasound guidance using a 21G needle. The patients were also advised to withhold NSAIDs two weeks prior and two weeks after injection. The patients were discharged 30 minutes post-injection and were advised to use ice packs multiple times/day to manage pain and abstain from strenuous exercises. Milder activities such as walking, basic strengthening exercises, and day-to-day activities were permitted.

Statistical analysis

Statistical analysis was performed using IBM SPSS software v22.0 (IBM Corp., Armonk, NY, USA). Due to variability in the number of patients at each follow-up time point (four to 2 months), scores at each follow-up compared to respective baseline scores were analyzed. For statistical analysis, the mean and standard deviation of WOMAC and Kujala scores were determined. Subgroup analysis involved patients suffering from grade I or II OA of the knee, males vs. females, and patients with either grade I or II OA of the knee with diabetes and/or hypertension. The Shapiro-Wilk normality test was performed, wherein the normal distribution of the data was assessed in order to determine continuation with the parametric statistical tests. A t-test was performed to determine differences between baseline and follow-up outcome measures scores and p<0.05 was considered significant (95% confidence interval). A repeated measures analysis of variance (ANOVA) was performed to determine differences between various follow-up time points and F-statistic value of <0.05 was considered significant. In case of the ANOVA test, Fisher's least significant difference (LSD) test was performed for multiple comparison tests and it was found that the 6th and 9th month follow-up time periods were found to be statistically significant as a correction of the p-value. The overall comparison data was found to be significant and the multiple comparisons showed deviations from significant results due to the difference in the number of individuals whose data was included in the study.

## Results

Leukocyte-poor platelet-rich plasma (LP-PRP) characterization

The data (mean ± standard deviation) obtained for LP-PRP characterization compared to the whole blood showed platelet concentration to be 6.79 times compared to the whole blood levels; the absolute platelet count to be 5.1 billion; and depletion of total leukocytes, lymphocytes, neutrophils, and RBCs by 88.66%, 82.34%, 98.48%, and 98.69%, respectively (Table 1).

**Table 1:** Leukocyte-poor platelet-rich plasma (LP-PRP) characterization compared to whole blood obtained via complete blood count (CBC) analysis.

	Whole blood (WB) (Mean ± SD)	Platelet-rich plasma (PRP) (Mean ± SD)	Leukocyte-poor platelet-rich plasma (LP-PRP) (Mean ± SD)	Results (LP-PRP versus WB)
Platelet count (10^3^/µL)	243±53.05	2049.33±102.17	1700.53±84.80	Platelet concentration: 6.79x; absolute platelet count: 5.1 billion; total leukocytes: depleted by 88.66%; lymphocytes: depleted by 82.34%; neutrophils: depleted by 98.48%; RBCs: depleted by 98.69%
White blood cells (WBCs)/total leukocytes (10^3^/µL)	6.44±1.01	10.36±7.43	0.73±0.53	
Lymphocytes (%)	34.77±3.62	76.8±4.62	6.14±0.32	
Neutrophils (%)	55.3±5.37	12.03±1.84	0.84±0.13	
Red blood cells (RBCs) (10^6^/µL)	3.83±0.34	0.12±0.07	0.05±0.03	

**Figure 2:**
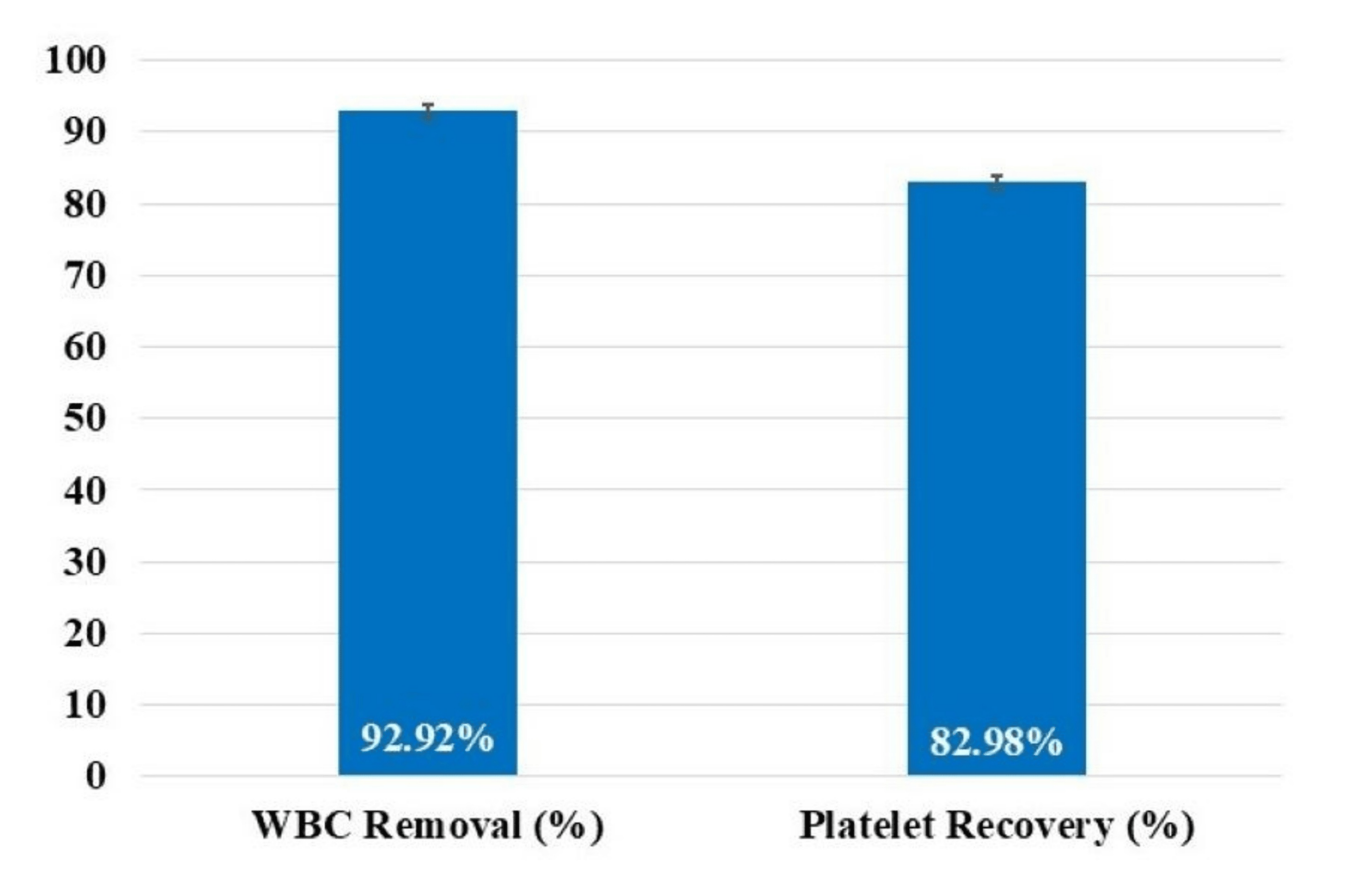
White blood cells (WBC)/total leukocytes removal and platelets recovery after passing formulated platelet rich plasma through PuriBlood leukocyte reduction filter (Puriblood Medical Co. Ltd., Taiwan).

In addition, about 93% of total leukocytes were removed and about 83% of the total platelets were recovered after passing formulated PRP through the PuriBlood leukocyte reduction filter (Puriblood Medical Co. Ltd., Taiwan) to obtain LP-PRP (Figure 2).

Patient characteristics

Patient characteristics of included patients, including age, gender, OA grade, affected knee and follow-up time points are summarized in Table 2.

**Table 2:** Patient demographics and characteristics.

Variables	Frequency (numbers)	Percentage (%)
Age (years)		
20-30	8	20
31-40	8	20
41-50	11	27.5
51-60	5	12.5
60-75	8	20
Gender		
Female	25	62.5
Males	15	37.5
Osteoarthritis grade (on the Kellgren-Lawrence scale)		
Grade I	20	50
Grade II	20	50
Affected knee		
Left	16	40
Right	23	57.5
Both	1	2.5
Follow-up time point		
4 months	4	10
5 months	4	10
6 months	12	30
9 months	17	42.5
10 months	2	5
12 months	1	2.5

Outcome measures

**Figure 3:**
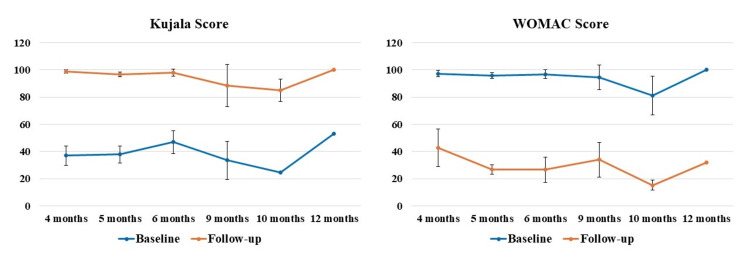
Statistically significant (p<0.05) differences were observed for both the Kujala and WOMAC scores at each follow-up time-point compared to the respective baseline scores. WOMAC, Western Ontario and McMaster Universities Osteoarthritis Index.

The results demonstrated statistically significant (p<0.05) differences in Kujala and WOMAC scores at each follow-up time-point compared to the respective baseline scores (Figure 3). Specifically, for Kujala score, an increase of 166.89%, 156.29%, 108.72%, 164.80%, 246.94%, and 88.68% were observed at 4, 5, 6, 9, 10, and 12 months follow-up, respectively, compared to the baseline. Similarly, for WOMAC score, a decrease of 56.26%, 72.18%, 72.62%, 64.13%, 81.34%, and 68.10% were observed at 4, 5, 6, 9, 10, and 12 months follow-up, respectively, compared to the baseline. No significant differences were seen when the differences in means at different follow-up times were compared to each other.

Subgroup analysis

**Figure 4:**
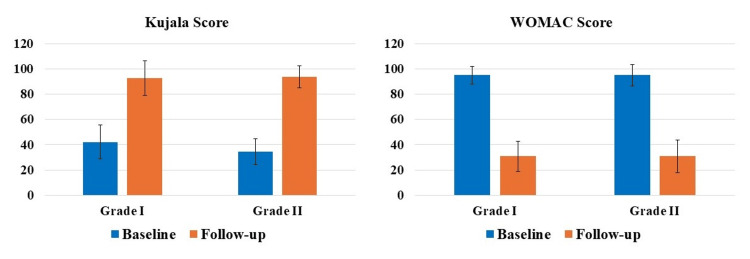
Difference between average baseline and follow-up Kujala and WOMAC scores for patients suffering with either grade I or II osteoarthritis of the knee was statistically significant (p<0.05). WOMAC, Western Ontario and McMaster Universities Osteoarthritis Index.

The subgroup analysis based on the grade of OA of the knee showed statistically significant (p<0.05) differences between the average follow-up Kujala and WOMAC scores compared to the baseline for patients suffering from either grade I or II knee OA (Figure 4). Specifically, patients with grade I OA showed a 120.43% increase in the Kujala score and a 67.6% decrease in the WOMAC score; whereas, patients with grade II OA showed a 171.74% increase in the Kujala score and a 67.53% decrease in the WOMAC score.

**Figure 5:**
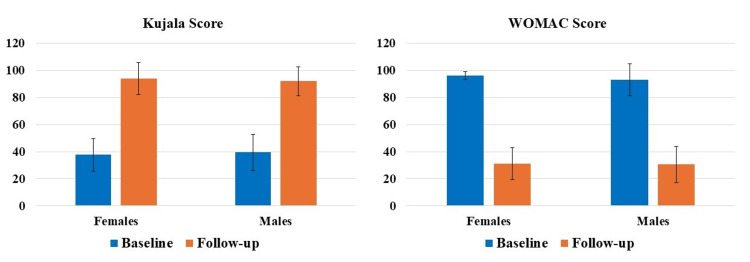
Difference between average baseline and follow-up Kujala and WOMAC scores for both males and females was statistically significant (p<0.05). WOMAC, Western Ontario and McMaster Universities Osteoarthritis Index.

In addition, the subgroup analysis based on gender showed statistically significant (p<0.05) differences between the average follow-up Kujala and WOMAC scores compared to the baseline for both females and males who participated in this investigation (Figure 5). Specifically, females showed a 149.89% increase in the Kujala score and a 67.82% decrease in the WOMAC score. Similarly, males showed a 133.45% increase in the Kujala score and a 67.13% decrease in the WOMAC score.

**Figure 6:**
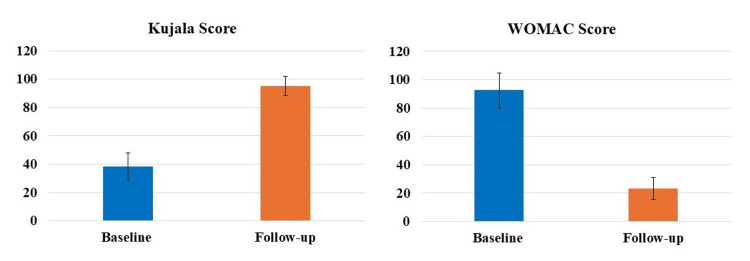
Difference between average baseline and follow-up Kujala and WOMAC scores for grade I or II knee osteoarthritis patients also suffering with diabetes was statistically significant (p<0.05). WOMAC, Western Ontario and McMaster Universities Osteoarthritis Index.

**Figure 7:**
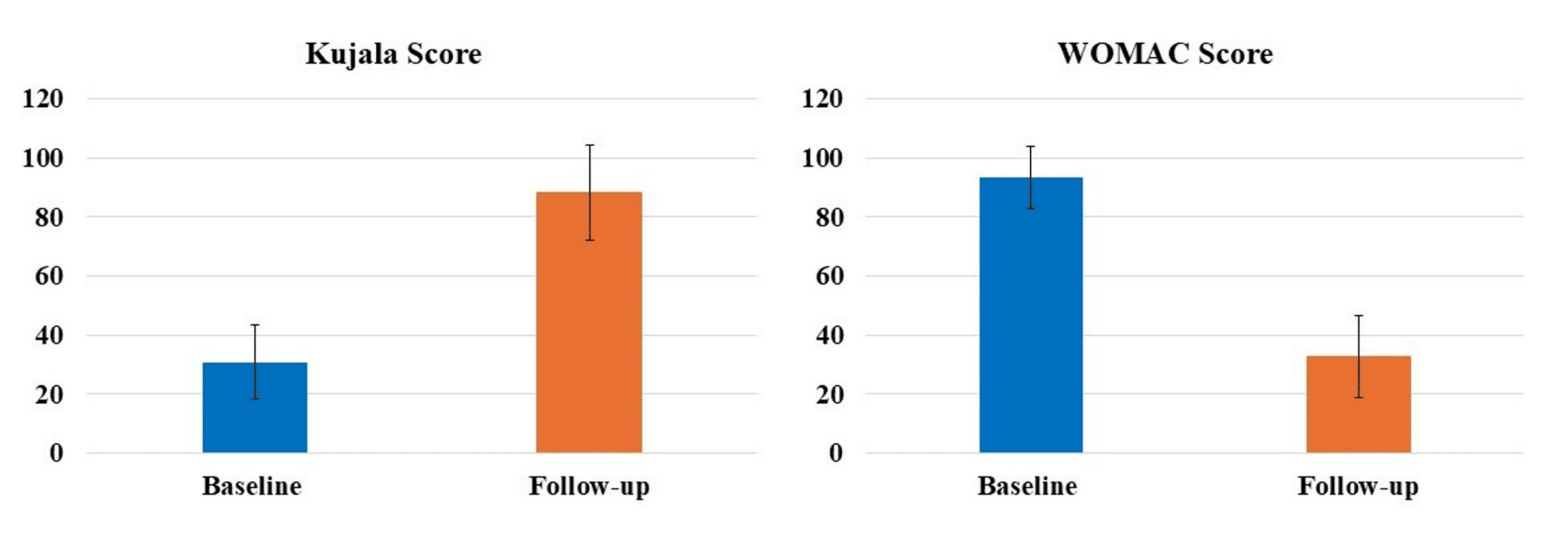
Difference between average baseline and follow-up Kujala and WOMAC scores for grade I or II knee osteoarthritis patients also suffering with hypertension was statistically significant (p<0.05). WOMAC, Western Ontario and McMaster Universities Osteoarthritis Index.

Moreover, some patients suffering from Grade I or II OA of the knee were noted to have other comorbidities, mainly hypertension and diabetes. Specifically, out of 20 patients with Grade I OA, 4 were suffering from hypertension; whereas, out of 20 patients with Grade II OA, six patients were suffering from hypertension, six patients from diabetes, and two patients with both hypertension and diabetes. The results from subgroup analysis showed statistically significant (p<0.05) differences between the average follow-up Kujala and WOMAC scores compared to the baseline for grade I or II knee OA patients also suffering from diabetes (Figure 6) and/or hypertension (Figure 7). Specifically, grade I or II knee OA patients suffering from diabetes showed a 147.01% increase in the Kujala score and a 74.87% decrease in the WOMAC score, while those suffering from diabetes showed 185.68% increase in the Kujala score and 64.78% decrease in the WOMAC score.

## Discussion

Orthobiologics including APBOs has emerged as a quickly progressive and innovative treatment modality in the field of orthopedic regenerative medicine, including for managing knee OA, focusing on the regeneration of damaged tissue, or delaying disease progression [11]. PRP is the most commonly used APBO; nonetheless, its effectiveness is debatable, due to the absence of standardized preparation protocol and characterization [7-11]. Literature has demonstrated that a platelet concentration of five to seven times compared to the baseline whole blood levels and platelet count of at least 5 billion is crucial for increasing cell migration and proliferation, tissue regeneration, and obtaining positive clinical outcomes [13,19-21]. In addition, pro-inflammatory neutrophils-led inflammation can worsen rather than ameliorate OA-associated pain [20]. Additionally, intra-articular injection of RBCs is detrimental, and they should be eliminated from PRP preparations [22]. In line with these, the results from our formulated LP-PRP demonstrated the platelet concentration to be at least six times compared to the baseline whole blood, the absolute platelet count to be at least 5 billion, and depletion of total leukocytes, lymphocytes, neutrophils, and RBCs by over 88%, 82%, 98%, and 98%, respectively (Table 1). Moreover, the PuriBlood leukocyte reduction filter (Puriblood Medical Co. Ltd., Taiwan) utilized in our study led to depletion of approximately 93% leukocytes and recovery of about 83% platelets (Figure 2). These results are in accordance with another study which compared two leukocyte reduction filters and reported >90% decrease in leukocyte count and >80% platelet recovery [18].

PROMs have achieved extensive relevance as healthcare continues to switch from volume to value, specifically as a vital sign of treatment excellence from the patient’s perception [23,24]. PROMs are enthusiastically favored by specialty organizations like the American Academy of Orthopaedic Surgeons, being progressively employed by foremost health coverage providers for musculoskeletal disorders [25,26]. PROMs measure particular elements of patients’ circumstances, including intensity of pain, limitations in activity, QoL, satisfaction with the treatment, etc. [25,26]. Though primarily applied in pharmacological research, recently the PROMs data has been increasingly utilized to evaluate the effectiveness of numerous treatments and care quality from a health policy and economics standpoint, being recognized as significant by the and the European Medicines Agency and United States Food and Drug Administration [27]. In our investigation, we used two widely used PROMs to assess knee, namely Kujala and WOMAC scores. Our results showed significant improvements in both Kujala and WOMAC scores at each follow-up time-point compared to the respective baseline scores in patients (both males and females) suffering from with either grade I or II OA of the knee (Figures 3-5). These results are in accord with the newly published randomized controlled trial(s) and systematic reviews and meta-analyses demonstrating the efficacy of LP-PRP for the treatment of knee OA [12,15,28,29]. This is then in harmony with the consensus from the European Society of Sports Traumatology, Knee Surgery and Arthroscopy (ESKAA) - ORthoBiologics InitiaTive (ORBIT), and ESKAA and International Cartilage Regeneration and Joint Preservation Society which recommends PRP use for managing grade I-III knee OA [30,31]. Interestingly, our subgroup analysis also showed significant improvements in both Kujala and WOMAC scores compared to the baseline score for grade I or II knee OA patients suffering with other comorbidities, specifically diabetes and/or hypertension (Figures 6, 7). Most clinical studies normally exclude patients with comorbidities; however, that is not the case in the real world day-to-day clinical practice. Thus, to accurately assess the effectiveness of LP-PRP in knee OA patients with hypertension and/or diabetes and to justify its routine clinical use, future studies with large sample size and longer follow-up are needed.

The presented research is not without shortcomings, including retrospective nature of the current investigation, small cohort size, short and varying follow-up duration, lack of radiographs or magnetic resonance imaging, and lack of commonly used treatment modalities for the management of knee OA, such as viscosupplementation, corticosteroids or other APBOs as comparators. Thus, more sufficiently powered, multi-center, prospective, non-randomized and randomized controlled trials with long-term follow-up are needed to establish the effectiveness of LP-PRP formulated using aforementioned leukodepletion filter in knee OA patients. Thus, all analysis and conclusions from this small sample retrospective single centre study are to be considered as exploratory and need further investigation.

## Conclusions

The intra-articular administration of formulated leukocyte-poor platelet-rich plasma (LP-PRP) resulted in statistically significant improvements in Kujala and WOMAC (Western Ontario and McMaster Universities Osteoarthritis Index) scores in both male and female grade I or II knee osteoarthritis patients with or without comorbidities, including diabetes and/or hypertension. The formulated LP-PRP demonstrated platelet concentration to be at least 6x compared to the baseline whole blood levels, the absolute platelet count to be at least 5 billion, and total leukocytes, lymphocytes, neutrophils, and red blood cells were depleted by over 88%, 82%, 98%, and 98%, respectively. In addition, the utilization of PuriBlood leukocyte reduction filter (Puriblood Medical Co. Ltd., Taiwan) led to the depletion of approximately 93% leukocytes and recovery of about 83% platelets.

## References

[1] Hunter DJ, March L, Chew M (2020). Osteoarthritis in 2020 and beyond: a Lancet Commission. Lancet.

[2] Azzini GO, Santos GS, Visoni SB, Azzini VO, Santos RG, Huber SC, Lana JF (2020). Metabolic syndrome and subchondral bone alterations: the rise of osteoarthritis - a review. J Clin Orthop Trauma.

[3] Deshpande BR, Katz JN, Solomon DH (2016). Number of persons with symptomatic knee osteoarthritis in the US: impact of race and ethnicity, age, sex, and obesity. Arthritis Care Res (Hoboken).

[4] Li D, Li S, Chen Q, Xie X (2020). The prevalence of symptomatic knee osteoarthritis in relation to age, sex, area, region, and body mass index in China: a systematic review and meta-analysis. Front Med (Lausanne).

[5] Aratikatla A, Maffulli N, Rodriguez HC, Gupta M, Potty AG, El-Amin SF 3rd, Gupta A (2022). Allogenic perinatal tissue for musculoskeletal regenerative medicine applications: a systematic review protocol. J Orthop Surg Res.

[6] Comlek S, Baykal D, Ozgonenel L (2024). Ultrasound-guided PRP and SVF therapy shows sustained improvement in severe knee osteoarthritis: a 12-month retrospective study. Med Sci Monit.

[7] Gupta A, Maffulli N (2024). Growth factor concentrate (GFC) for the management of osteoarthritis of the knee: a systematic review. Indian J Orthop.

[8] Gupta A, Jain V (2024). Autologous conditioned plasma (ACP) and osteoarthritis of the knee: a review of current clinical evidence. Cureus.

[9] Gupta A, Aratikatla A (2024). Hyperacute serum and knee osteoarthritis. Cureus.

[10] Gupta A (2024). Autologous protein solution (APS) and osteoarthritis of the knee: a scoping review of current clinical evidence. Cureus.

[11] Gupta A, Jain VK (2024). Autologous peripheral blood-derived orthobiologics: different types and their effectiveness in managing knee osteoarthritis. World J Orthop.

[12] Xiong Y, Gong C, Peng X (2023). Efficacy and safety of platelet-rich plasma injections for the treatment of osteoarthritis: a systematic review and meta-analysis of randomized controlled trials. Front Med (Lausanne).

[13] Patel S, Gahlaut S, Thami T, Chouhan DK, Jain A, Dhillon MS (2024). Comparison of conventional dose versus superdose platelet-rich plasma for knee osteoarthritis: a prospective, triple-blind, randomized clinical trial. Orthop J Sports Med.

[14] Gupta A, Jeyaraman M, Potty AG (2023). Leukocyte-rich vs. leukocyte-poor platelet-rich plasma for the treatment of knee osteoarthritis. Biomedicines.

[15] Chen L, Jin S, Yao Y, He S, He J (2023). Comparison of clinical efficiency between intra-articular injection of platelet-rich plasma and hyaluronic acid for osteoarthritis: a meta-analysis of randomized controlled trials. Ther Adv Musculoskelet Dis.

[16] Jayaram P, Liu C, Dawson B, Ketkar S, Patel SJ, Lee BH, Grol MW (2020). Leukocyte-dependent effects of platelet-rich plasma on cartilage loss and thermal hyperalgesia in a mouse model of post-traumatic osteoarthritis. Osteoarthritis Cartilage.

[17] Di Martino A, Boffa A, Andriolo L (2022). Leukocyte-rich versus leukocyte-poor platelet-rich plasma for the treatment of knee osteoarthritis: a double-blind randomized trial. Am J Sports Med.

[18] Cho JH, Choi JH, Hur M, Moon HW, Park CM, Yun YM (2012). Comparison of two leukocyte reduction filters for whole blood derived platelets. Transfus Apher Sci.

[19] Berrigan WA, Bailowitz Z, Park A, Reddy A, Liu R, Lansdown D (2024). A greater platelet dose may yield better clinical outcomes for platelet-rich plasma in the treatment of knee osteoarthritis: a systematic review. Arthroscopy.

[20] Mautner K, Malanga GA, Smith J, Shiple B, Ibrahim V, Sampson S, Bowen JE (2015). A call for a standard classification system for future biologic research: the rationale for new PRP nomenclature. PM R.

[21] Giusti I, Rughetti A, D'Ascenzo S, Millimaggi D, Pavan A, Dell'Orso L, Dolo V (2009). Identification of an optimal concentration of platelet gel for promoting angiogenesis in human endothelial cells. Transfusion.

[22] Gupta A, Maffulli N, Jain VK (2023). Red blood cells in platelet-rich plasma: avoid if at all possible. Biomedicines.

[23] Norwood SM, Han D, Gupta A (2024). H-Wave(®) device stimulation for chronic low back pain: a patient-reported outcome measures (PROMs) study. Pain Ther.

[24] Gupta A, Han D, Norwood SM (2024). H-Wave(®) device stimulation for chronic neck pain: a patient-reported outcome measures (PROMs) study. Pain Ther.

[25] Lowry KJ, Brox WT, Naas PL, Tubb CC, Muschler GF, Dunn W (2019). Musculoskeletal-based patient-reported outcome performance measures, where have we been-where are we going. J Am Acad Orthop Surg.

[26] Plate JF, Deen JT, Deans CF, Pour AE, Yates AJ, Sterling RS (2024). Implementation of the new medicare-mandated patient-reported outcomes after joint arthroplasty performance measure. J Arthroplasty.

[27] Kluzek S, Dean B, Wartolowska KA (2022). Patient-reported outcome measures (PROMs) as proof of treatment efficacy. BMJ Evid Based Med.

[28] Wang R, Xie Y, Xie L, Liu J, Jia J, Chen X, Wu Q (2024). Platelet-rich plasma versus corticosteroid in the treatment of knee osteoarthritis: a systematic review and meta-analysis of randomized controlled trials. Georgian Med News.

[29] Yoshioka T, Arai N, Sugaya H (2024). The effectiveness of leukocyte-poor platelet-rich plasma injections for symptomatic mild to moderate osteoarthritis of the knee with joint effusion or bone marrow lesions in a Japanese population: a randomized, double-blind, placebo-controlled clinical trial. Am J Sports Med.

[30] Laver L, Filardo G, Sanchez M (2024). The use of injectable orthobiologics for knee osteoarthritis: A European ESSKA-ORBIT consensus. Part 1-blood-derived products (platelet-rich plasma). Knee Surg Sports Traumatol Arthrosc.

[31] Kon E, de Girolamo L, Laver L (2024). Platelet-rich plasma injections for the management of knee osteoarthritis: the ESSKA-ICRS consensus. Recommendations using the RAND/UCLA appropriateness method for different clinical scenarios. Knee Surg Sports Traumatol Arthrosc.

